# Measles: An Updated Literature Review of the Host Response, Pathogenesis, Complications, Prevention Measures, and Recent Outbreaks

**DOI:** 10.3390/cimb48020206

**Published:** 2026-02-13

**Authors:** Stefanie Au, Seema Saini, William Dela Cruz, Vishwanath Venketaraman

**Affiliations:** College of Osteopathic Medicine of the Pacific, Western University of Health Sciences, Pomona, CA 91766, USA; stefanie.au@westernu.edu (S.A.); seema.saini@westernu.edu (S.S.); william.delacruz@westernu.edu (W.D.C.)

**Keywords:** measles, rubeola, *Paramyxoviridae*, RNA virus

## Abstract

Measles remains a significant global health threat due to its extreme transmissibility and the potential for severe, long-term complications. This review synthesizes the most up-to-date literature on the host response, immunological impact, current treatments, and prevention of the measles virus (MeV). The review details host immune evasion mechanisms, including the antagonism of interferon signaling, discusses genetic associations with vaccine failure, and explores adjunctive treatments like vitamin A and ribavirin. Despite the success of the two-dose MMR vaccine, recent resurgences in the United States, peaking at 2065 cases in 2025, underscore a critical decline in herd immunity driven by vaccine hesitancy and pandemic-related disruptions. However, with no FDA-approved antiviral, primary prevention via vaccination remains the only effective strategy. This paper emphasizes the necessity of strengthening public health outreach and standardizing international surveillance to mitigate the rising incidence of this preventable disease.

## 1. Introduction

Measles, also known as Rubeola, is a highly contagious, potentially fatal, febrile disease that arises from infection with measles virus (MeV), a negative-sense, non-segmented, single-stranded RNA virus within the *Paramyxoviridae* family. The viral RNA encodes six structural genes, including the phosphoprotein (P) gene, hemagglutinin (H) protein gene, and fusion (F) protein gene [1]. Host target cells have been found to express the signaling lymphocytic activation molecule (SLAM; also known as CD150) and/or nectin-4, which are found on immune cells and respiratory epithelial cells, respectively. Infection is initiated when the H protein attaches to the host cell and induces a conformational change within the F protein that promotes the release of viral cell products into the host cell [2]. The viral genome is replicated and expressed within these target cells, which subsequently express the H and F proteins on the surfaces of infected cells. Multinucleated giant cells are then formed through the fusion of infected neighboring cells. Infection is commonly disseminated throughout the body through direct cell-to-cell transmission of infected lymphocytes via intercellular pores [1]. More specifically, infected dendritic cells (DCs) and macrophages further transmit the infection to mononuclear cells such as thymocytes, B cells, and hematopoietic stem cells, which are responsible for dissemination to non-lymphoid organs [3]. Evidence of MeV-associated immune suppression is demonstrated by declining numbers of CD4+ T cells, CD8+ T cells, and B cells. This notable lymphopenia leads to immune suppression and increased susceptibility to opportunistic infections [1]. The consequences of measles-associated immune suppression last for many years after initial infection and lead to increased mortality and morbidity. The virus promotes the destruction of memory T and B cells against other infectious agents and naive B cells that express CD150 [4]. This alteration in lymphocytes leads to an imbalance of immune cells and a reduced humoral immune memory that can last months to years [2]. (See also “Section 4. Host Response”).

The prodromal phase of MeV infection includes a cough and fever. The onset of fever above 38.3 °C (101 °F) and the maculopapular rash occurs about 10 and 14 days after initiation of MeV infection, respectively [5]. Infected persons are likely the most contagious between 4 days before and 4 days after the onset of the pathognomonic morbilliform rash [1], when viral titers are at their highest concentrations in the respiratory tract. Evidence of the rash is absent in immunocompromised patients, implying that emergence of a rash indicates the initiation of the host immune response [5].

Transmission of this virus predominantly occurs via inhalation of infectious aerosols and leads to characteristic symptoms including fever, coughing, coryza, conjunctivitis, and Koplik spots [1]. It is often assumed that a single infected case can lead to 12–18 secondary cases [2], but this average can vary depending on other variables. These covariates include socioeconomic factors that influence the number of contacts an infected person may have, such as population density and the birth rate [6]. Additionally, accessibility and utilization of the measles vaccine influence the rate of transmission. Populations at greater risk of high transmissibility include those within densely populated urban spaces who concurrently experience low vaccination rates [7]. Higher-income nations have an estimated coverage of 94% and 91% for the first and second doses of the measles vaccine, compared to a worldwide coverage of 85% and 67%, respectively [4]. Furthermore, the risk of measles-related complications, including mortality, increases in those experiencing a poor nutritional status and/or immune compromise. For example, the nutritional status plays a role in providing metabolic power for mounting an immune response against the infection. More specifically, vitamin deficiencies are common in individuals with a poor nutritional status such as vitamin A deficiency, which is linked to ophthalmologic complications including keratitis, corneal scarring, and blindness [1]. While at-risk groups primarily include infants and unvaccinated adolescents, infections acquired by unvaccinated adults without prior immunity can also lead these adults to present with severe illness [8,9].

While the United States (U.S.) declared measles eliminated in 2000, the exponential increase in caseloads within the past twelve years provides concerning evidence that the U.S. is experiencing dwindling herd immunity. Consequently, this rise has led to an increase in measles-related research. While the current literature offers robust evidence of the epidemiology of measles, there are limited works that provide a comprehensive review of the immunologic impact of a measles virus infection. In addition to summarizing the epidemiology of measles, this literature review aims to consolidate current research findings with a refined lens and focus on the immunological consequences of MeV infection, genetic considerations for infection susceptibility, prevention measures to mitigate the risk, and treatment recommendations to prevent long-term sequelae.

## 2. Materials and Methods

A literature search was conducted in the PubMed, Medline, CINAHL, and Google Scholar databases using algorithms with terms related to measles, also known as Rubeola. Titles and abstracts were screened by three independent reviewers to identify clinical studies published between 2016 and 2025 that examined the immune response, pathogenesis, increased incidence, and prevention of measles. Additional studies not captured in the initial database search from review articles were also assessed in this review. Clinical trials, interventional studies, retrospective chart reviews, systematic reviews, case series, and individual case reports were included in the literature review. The findings from these publications were synthesized narratively, with a focus on the mechanisms of the host immune response and recent outbreaks in the U.S. No generative artificial intelligence (GenAI) has been used in this paper.

## 3. Background and Epidemiology

### 3.1. Historical Context (1600s–1990s)

While measles has been around for centuries, the highly contagious and potentially fatal illness was not distinguished from scarlet fever and rubella until the 1600s and late 1800s, respectively. By 1918, a British doctor, J.S. Warrack, characterized measles, scarlet fever, and rubella as distinct illnesses. Lutheran Church records in Finland from 1700 to 1800 provide written evidence of lethal epidemic cycles with symptoms that resemble what is now known about MeV infection [10].

In the U.S., epidemic cycles of measles ultimately caused all persons to endure a wild-type measles infection before reaching adulthood. In the 20th century, an estimated half a million individuals were infected with the virus annually, with one in every thousand infected persons dying from MeV. Additionally, about 1000 measles patients suffered permanent brain damage from measles-related complications [11].

By 1963, a one-dose measles vaccine was implemented, and by 1970, measles cases dropped below one hundred thousand. The following year, a combination vaccine for Measles, Mumps, and Rubella (MMR) was introduced. The greatest impact of vaccine protection was observed in school-aged children; therefore, by 1989, the Advisory Committee on Immunization Practices (ACIP) introduced a two-vaccine protocol specifically for this age group. Some children remained unvaccinated despite the proven efficacy of vaccines, which led to outbreak cases between 1989 and 1991 [11].

### 3.2. United States Eradication and Outbreaks (2000–Present)

Globally, widening immunity gaps and disrupted healthcare systems have contributed to a measles resurgence that has reached alarming levels in recent years. According to World Health Organization (WHO) surveillance data, global measles cases were estimated at 11 million in 2024, an 8% increase compared to 2019 levels before the pandemic. While the WHO African Region reported a 40% decrease in cases in 2024, the disease burden notably shifted toward middle-income countries and regions facing geopolitical instability. Nonetheless, there are disproportionately higher rates of measles cases in fragile, conflict-affected, and vulnerable settings than in non-conflict settings. This disparity is exacerbated by war-related migration and the collapse of routine immunization infrastructure [12,13].

In contrast to the trends in some low-income regions, Europe has experienced a sharp resurgence that is largely attributable to vaccine hesitancy. Data from the European Centre for Disease Prevention and Control (ECDC) reveals that the EU/EEA reported over 35,000 measles cases in 2024. This rise marks a tenfold increase from the previous year and exceeds figures from 2019 [14]. The resurgence in Europe provides evidence of the volatility of measles control in high-resource areas when vaccination coverage falls below the elimination threshold.

In analyzing trends within the United States, vaccination programs proved to be successful, and by 2000, measles was declared eliminated per the Centers for Disease Control (CDC) website, as MeV cases in 2000 reached below 100. Any new cases after this time point resulted from imported cases from travelers. In alignment with this elimination status, vaccination recommendations in the U.S. included administration of the first dose between 12 and 18 months of age and the second dose between 4 and 6 years old. While caseloads fluctuated from 2000 to 2025, confirmed positive cases remained below 250 until 2014. Notably, cases reached 667, 1274, and 2065 during 2014, 2019, and 2025, respectively [12].

Despite its elimination status, unvaccinated persons and those with an unknown vaccination status have accounted for 88% of the confirmed measles cases in the U.S. since 2001 [15]. Since the COVID-19 pandemic, the decline in vaccination rates and public mistrust in vaccination measures have further perpetuated the potential for a resurgence of measles infections within the U.S. [13]. As a result, first-dose measles vaccine coverage dropped below 81%, the lowest it had been since 2008. While the vaccination rate began to rise again in 2022, the rate plateaued at 83% and remained unchanged in 2023 [15,16].

**Figure 1:**
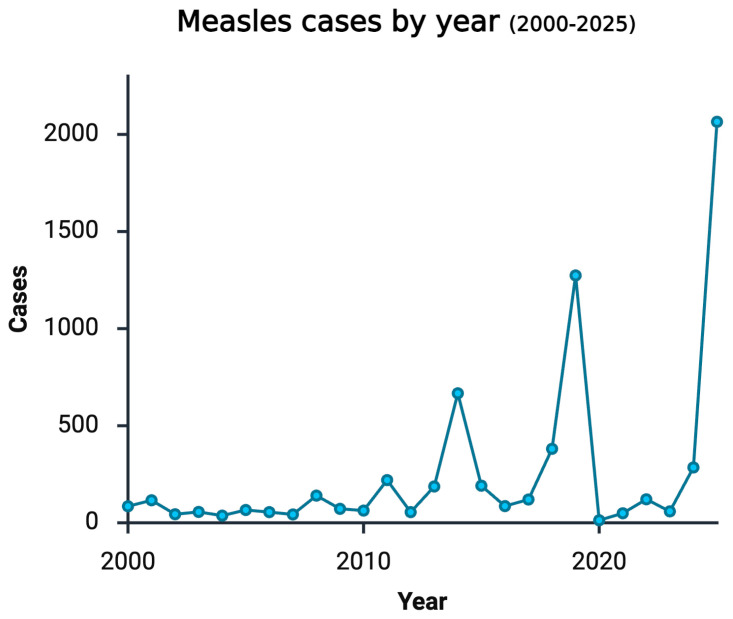
Line graph depicting the incidence of measles cases per year from 2000 to 2025, highlighting the fluctuations and the overall sharp increase from 2010 to 2025 per the CDC website. Created in BioRender. Au, S. (2026) BioRender.com/43mk7qx.

Ultimately, reduced vaccination rates and vaccine hesitancy laid the groundwork for a measles resurgence in the U.S. (Figure 1). Among measles cases analyzed by the CDC from 1 January to 17 April 2025, 800 cases were confirmed. The CDC was unable to classify cases in Texas as vaccinated versus unvaccinated during this period due to a specific opt-in law, so the 590 Texas cases were excluded from analysis. In the 210 remaining cases, 77% were unvaccinated, 3% had received one MMR dose, 6% had received two MMR doses, and 14% had an unknown vaccination status [16,17]. Evidence of this recent resurgence displays the necessity of widespread vaccine coverage of the measles virus.

Collectively, the comparison of recent U.S. outbreaks against broader global trends highlights a critical commonality: measles exploits immunity gaps regardless of a region’s resource level. While the specific drivers can range from vaccine hesitancy in high-income nations to infrastructural collapse in conflict-affected zones, the trajectory of resurgence is uniform. These findings provide clear evidence that sporadic interventions are insufficient; sustaining widespread vaccination coverage above the 95% elimination threshold remains the singular key to controlling and eventually eliminating this destructive disease.

## 4. Host Response

### 4.1. Host Response and Virus Immune Evasion

Several mechanisms unique to MeV demonstrate why measles remains an ongoing public health threat without adequate treatments to combat it. Some of the main characteristics associated with MeV infection are its ability to evade multiple pro-inflammatory cytokine responses, including IFN signaling, the capacity to block various downstream signaling pathways, and the overall depletion of B- and T-cell lymphocytes. These evasion mechanisms allow MeV to downregulate innate immune responses.

Infection of immune cells by MeV occurs by CD150-presenting dendritic cells and macrophages detecting its viral RNA. Dendritic cells are typically central to the body’s innate immune response, as downstream signaling cascades produce pro-inflammatory cytokines such as interleukin-12 (IL-12), a key factor in the priming and differentiation of interferon gamma (IFNγ)-producing CD4+ and CD8+ T cells. However, MeV’s viral N protein engages with FcγR molecules on DCs, and MeV’s viral H protein engages with CD46, effectively reducing IL-12 production and resulting in a state of lower immunity. Moreover, viral hemagglutinin H as well as other MeV gene products attack many steps of IFN induction and the IFN signaling cascade. By interacting with and infecting immune cells, MeV alters immune cell functions, leading to impaired T cell and antibody responses [18]. Additionally, MeV’s P, V, and C proteins antagonize the IFN system. The exact mechanism of how it happens is not yet that clear, but it is thought that the V protein interferes with STAT1 and STAT2, both essential proteins in the IFN system [19].

Recent evidence depicts the importance of RNA from “defective interferant” (DI) subgenomic particles in the activation of cytoplasmic RIG-I-like receptors (RLRs). RLRs are typically involved in the detection of infected cells; however, MeV’s C protein has been described as altering viral replication by diminishing the levels of DI RNA, controlling cell death and innate immunity pathways, and blockading PKR downstream signaling involving the NF-κB, IRF-1 and MAPK pathways [20].

The last way in which MeV evades the immune system is the depletion of lymphocytes. Downregulation of IL-12 already limits CD4+ and CD8+ T-cell production; however, exposure of MeV to mucosal-associated invariant T (MAIT) cells, a subset of lymphocytes, results in subsequent apoptosis [20]. As early as 1 h after MeV inoculation, the majority of infected MAIT cells were in early stages of apoptosis. By depleting MAIT cells, it is likely that MeV induces other mucosal infections, which results in an immunosuppressed state [21].

Besides its many mechanisms to evade both the innate and adaptive immune systems [Table 1], MeV is unique in its ability to cause complications months to years after the initial infections. Long-term consequences of MeV are abundant, and many of the resulting consequences occur due to loss of adaptive immunity to previously exposed pathogens and depletion of B memory clones.

### 4.2. RNA Sequencing Studies

Recent analysis has unveiled which pharyngeal transcriptomes are involved in host responses to MeV. Analysis of the pharyngeal epithelium transcriptome revealed several key genes known to be involved in host immune responses. Specifically, two upregulated genes, *RAD2* and *OASL*, known to respond to viral infection, were observed. The *IFIH1 (MDA-5)* gene and several IFN-induced genes (*IFIT3*, *IFIT2*, *IFIT1*, *IFITM3*, *IFI44*), shown to trigger the release of interferons (IFNs), were also upregulated. Innate immunity activation was also depicted by the increased expression of *CLEC7A (DECTIN 1)*, *CLEC4E*, and *TNFAIP6* genes. Finally, overexpression of chemokines *CXCL1* and *CXCL2* indicates the presence of inflammation in the pharynx, causing NLRP3 inflammasome induction in macrophages. The *AIM2* gene was upregulated, allowing the assumption that dsDNA-dependent AIM2-inflammasome activation is successful, leading to AIM2-mediated IL-1β and IL-18 production [22].

Like other known viral pathogens, MeV carries components that mimic regulatory elements of the host cells, enabling MeV to intervene in essential human immune processes. Moreover, another upregulated gene, *IVNS1ABP (NS-1)*, is known to play a role in apoptosis inhibition, DC maturation suppression, as well as control of protein stability, and acts as a regulator of transcription of host cell mRNAs. NS-1 also has the ability to act as a histone mimic with its H3-like sequence, allowing it to suppress antiviral genes’ expression. Overexpression of *TNFAIP3/A20*, a gene that acts as an inhibitor of both NFκB activation and TNF-mediated apoptosis, was also observed [22].

### 4.3. Genetic Associations with Failed Immunity

Despite an available vaccine, MeV remains an important re-emerging pathogen with a continuous increase in prevalence worldwide during the last decade. At present, none of the WHO’s measles elimination goals have been met. Some patients may be hesitant due to MMR vaccines’ potential adverse effects and PVF; however, recent studies have discovered CD46 and IFI44L genetic variants to be determinants of measles vaccine-induced humoral immunity and post-vaccination adverse events. Although primary and secondary vaccine failure are not uncommon, SVF MeV infections tend to have a less severe symptomology and complications [23].

A genome-wide single-nucleotide polymorphism (SNP) genotyping study on MeV vaccination efficacy demonstrated that four SNPs (rs3005891, rs77498152, rs79225096, rs4474098) correlated with the vaccine response. Furthermore, their study found that for measles PRNT, the four-digit HLA type with the strongest association was HLA-A2902, which was negatively associated with the measles PRNT outcome (*p* = 0.006). The strongest association was found for measles IgG at HLA-B1801 (*p* = 0.0025) [24]. Together, these studies suggest that a genetic predisposition plays a large role in the vaccine response.

### 4.4. Oxidative Stress

As discussed, MeV leads to fatal complications such as SSPE and immune depletion (See more: “Section 4.5. Complications”). These fatal complications have led researchers to discover risk factors associated with an increased incidence of SSPE, such as vitamin A deficiency and low levels of antioxidants. While the mechanisms remain unclear, it can be concluded that vitamin A deficiency and low levels of antioxidants are related to underlying oxidative stress, impairing the human immune response and increasing the body’s susceptibility to infections. Furthermore, these immune deficiencies potentiate excessive inflammation and thus contribute to the severe life-threatening effects of MeV [25].

The human body has naturally occurring antioxidants to fight off oxidative stress, including compounds such as the thiol group, also known as mercaptan. Thiols make up a large component of naturally occurring antioxidants and are involved in many cellular functions, including defense, programmed cell death, and enzyme regulation. A study conducted in Turkey concluded that the total antioxidant status value in children infected with MeV was significantly lower, while the total oxidant status and oxidative stress index values were significantly higher (*p* < 0.05) compared to the control group [26]. This suggests that the antioxidant status is inversely related to measles susceptibility, and that antioxidants are important in controlling infection. However, more insights are needed to determine whether antioxidant supplementation can improve measles outcomes and overall prevent viral infection (See more “Section 4.6.2. Supplementation”).

### 4.5. Complications

Measles is associated with many complications, which can be organized into three categories: (i) acute, including diarrhea, otitis media, pneumonia, encephalitis, seizures, and death, (ii) delayed SSPE, and (iii) post-measles immune amnesia [27]. Complications are more likely to occur in children under 5 years old, adults over 20, and pregnant, immunocompromised, or malnourished patients [2]. Additionally, vitamin A deficiency has been linked to higher rates of MeV infection and subsequent complications [28] (see more “Section 4.6.2. Supplements”).

#### 4.5.1. Acute Complications

Acute complications of MeV infection include pneumonia, keratoconjunctivitis, otitis media, and diarrhea. The most common complication is acute otitis media, while the most severe complications are pneumonia and laryngotracheobronchitis, respectively [29]. Keratoconjunctivitis has also been noted to be present in the early stages of measles infection and can persist for up to 3 months after onset. Measles-related otitis media has been found to cause sensorineural deafness, which was most prominent in the pre-vaccination era of measles [30].

Fatal complications of measles include acute postinfectious measles encephalitis, measles inclusion body encephalitis (MIBE), and SSPE. Acute postinfectious measles encephalitis can present within the first week of symptoms and is associated with a 20% mortality rate. In contrast, MIBE presents within 6 months of symptom onset and is associated with a 100% mortality rate. Finally, subacute sclerosing panencephalitis (SSPE) is the most progressive complication of measles. SSPE presents 7–10 years after initial infection and causes a 100% mortality rate within 1–3 years of onset [30] (see more “Section 4.5.3. Encephalitis”).

#### 4.5.2. Measles-Associated Pneumonia (MAP)

The most common complication that leads to hospitalization and possible death within unvaccinated children is measles-associated pneumonia (MAP), which occurs in 1 in 20 children infected with MeV [3,27]. Pneumonia from secondary infections and coinfections are common, as MeV-induced immune dysregulation leaves infected patients susceptible to many pathogens [31]. However, pneumonia is not the only possible lung complication. In a 2020 retrospective study, 16% (28) of patients with measles developed pleural effusions and 10% (17) of patients developed pneumothorax. Five patients went on to develop acute respiratory distress syndrome (ARDS), and two died from it. In this study, 99% (175) of patients hospitalized for measles were unvaccinated [32].

In pregnant patients, measles poses a risk to both the mother and unborn fetus. While measles is not associated with a congenital syndrome, MeV can cause spontaneous abortion, premature labor, and a low birth weight. Additionally, respiratory distress in the fetus and/or mother as well as maternal deaths in unvaccinated mothers have also been widely documented [33].

#### 4.5.3. Encephalitis

Neurological complications of measles are uncommon but often lead to serious disability or death. Acute disseminated encephalomyelitis, also known as acute postinfectious encephalomyelitis, is an inflammatory autoimmune demyelinating disease of the CNS. This encephalomyelitis occurs in approximately 1 in 1000 people with measles, often occurring within 2 weeks of infection. This autoimmune disease is frequently fatal, and if not, can have life-lasting consequences, including cognitive deficits, motor deficits, and visual impairment [34].

In immunocompromised patients, a risk of MIBE, also known as subacute measles encephalitis, has been documented in 49 patients [35]. MIBE tends to occur 1–9 months after the initial measles infection and has been defined as subacute progressive encephalopathy rapidly culminating in death. The literature on MIBE is limited, but the mechanism is likely related to the cell-to-cell spread of mutated neurotropic measles viruses. The only way to diagnose MIBE has been through biopsy, in which histology will demonstrate a moderate increased cellular density and absence of nuclear inclusion bodies [36]. In the 49 patients, more than two-thirds of them either were unvaccinated or their vaccination records were unverified [35]. This suggests a higher risk of fatal complications such as MIBE in unvaccinated patients.

On the other hand, SSPE is a persistent brain infection that can occur up to 20 years after the initial measles infection [27]. It is more likely to occur with a younger age of measles onset, leads to motor dysfunction, personality changes, and eventual seizures and death. The risk of SSPE is 1 per 1000 [2]. Patients are often immunocompetent and have a period of latency of around 5–10 years. The mechanism of how MeV manages to spread to the brain is unknown, and MeV has not been found in cultures of SSPE patients [35].

#### 4.5.4. Immune Amnesia

During measles infections, transient lymphopenia occurs due to the migration of the lymphocytes from the bloodstream into the lymphatics where MeV tends to proliferate. It has been proposed that IL-10 levels remain elevated for weeks following infection, resulting in further immunosuppression [19]. Despite lymphocyte counts frequently recovering shortly after the appearance of the measles-associated rash, immunosuppression can persist for months to years after infection. B-cell receptor sequencing allowed the identification of incomplete reconstitution of the naïve B-cell pool and compromised immune memory to previously encountered pathogens due to the depletion of previously expanded B memory clones [37]. Another study demonstrated substantial reductions in the number of pathogen epitopes recognized after measles, resulting in a loss of antibody diversity compared to unexposed controls [38].

In terms of T lymphocytes, MeV affects T-cell function directly during the active phase of measles infection. Through the interaction of MeV H and proteolytically activated F with lipid rafts on the lymphocyte cell surface, MeV can block cell cycle progression. In all, MeV acts both directly on T cells to inhibit activation-induced proliferation and produces a long-term generalized effect on pathogen recognition, often lasting years [39]. Together, these recent studies show that the interaction between MeV and CD-150 DCs induces a depletion of B- and T-lymphocyte subsets, loss of B memory cell clonal diversity, and depletion of antibody production [5,37,38,40].

### 4.6. Treatments and Supplements

Currently, there remains no antiviral therapy to treat measles, and prevention is the mainstay of disease control worldwide. Measles has a long incubation period before viremia appears, and thus, there is adequate time to introduce antiviral therapies. This requires more clinical trials in areas with measles outbreaks to attempt various types of rapid antiviral therapy with the goal of completely preventing infection onset in naive patients [19].

#### 4.6.1. Post-Exposure Prophylaxis (PEP)

Two post-exposure prophylaxis (PEP) treatments have been studied in the prevention of measles and its complications, including immunoglobulins (Ig) and the MMR vaccine. However, PEP is only considered in certain countries, especially if live vaccines are contraindicated [2]. The effectiveness of Ig PEP has ranged from 76% (95% CI 0–94) to 100% (95% CI 56.2–99.8%), while that of MMR PEP has ranged from 83.4% (95% CI 34.4–95.8) to 100% (95% CI not estimable) [41]. Out of two studies, one including 33 children and one including 7 neonates, researchers found that the patients did not have any post-measles complications after the administration of Ig PEP. In addition, Ig PEP has been well-tolerated, with an infrequent complication of self-limiting grade 1 fevers in 3.2% of participants [42]. Studies following the PEP administration of the MMR vaccine demonstrated that compared to controls, military recruits with PEP had shorter hospitalizations (8.3 days [SD 4.0] compared to 13.9 days [SD 5.8]), a shorter duration of high fever (≥39 °C) (4.0 days [SD 3.2] compared to 6.7 days [SD 4.4]), a lower maximum fever temperature (39.3 °C [SD 0.9] compared to 40.4 °C [SD 0.6]), and fewer cases of acute conjunctivitis (38% compared to 100%) [41]. While promising, the reports on effective measles PEP are limited in number, with reports on different age groups, adjunctive treatments, and larger studies lacking control groups.

#### 4.6.2. Supplementation

The WHO recommends that patients with measles who are younger than 5 years receive one dose of vitamin A at their first presentation to the health facility. This is then followed by a second dose 1 day later. If any signs of vitamin A deficiency are observed, such as xerophthalmia, Bitot’s spots, and corneal ulceration, a third dose should be administered 4–6 weeks later. This is due to the association of MeV infection with low circulating retinol levels. These reduced levels in the bloodstream are likely caused by inflammation related to measles, which disrupts vitamin A transport. Systemic inflammation and hepatic dysfunction lead to reduced synthesis of retinol-binding protein, resulting in vitamin A accumulating in the liver as well [43,44]. However, clinical manifestations of measles, including anorexia, nausea, diarrhea, and dry skin, overlap with symptoms of vitamin A toxicity, suggesting that measles involve dysregulated hepatic retinoid metabolism with abnormal release of stored retinyl esters into tissue. In this context, supplementation of vitamin A appears to go beyond correcting deficiency and may involve modifying vitamin A metabolism and mobilization, including stabilizing the retinol distribution and mitigating tissue damage and inflammation associated with measles [43].

Glutathione (GSH) is a cysteine-containing tripeptide that serves as a natural antioxidant. GSH and its derivatives are antioxidant substances that play many roles in the body’s homeostatic mechanisms, including apoptosis, enzymatic regulation, and detoxification. In the recent literature, mitochondria-mediated oxidative stress has been highlighted as a reason for reactive oxygen species (ROS) abundance, which causes cellular dysfunction and ultimately cell death. Virus-induced mitochondrial ROS (mtROS) benefit the viral life cycle, and viruses can exploit the cell’s mitochondria and ultimately control the host cell’s oxidative status. Due to GSH’s importance in reducing ROS production, thiol supplementation has been proposed as a promising antiviral strategy [45]. Furthermore, a recent study demonstrates the possibility of using thiol/disulfide homeostasis as oxidative stress markers in pediatric patients infected with measles [26]. Findings have even shown the presence of oxidative damage in pediatric patients with SSPE. Some common antioxidants, such as serum alpha-tocopherol, beta-carotene, retinol, ascorbic acid levels, and erythrocyte and cerebrospinal fluid, reduced GSH concentrations in SSPE patients [46].

Furthermore, vitamin D has been implicated in reducing oxidative stress in other RNA virus infections, such as COVID-19. A large-scale study also showed the inverse relationship between serum vitamin D levels and MeV antibody titers [47], suggesting that vitamin D supplementation could ameliorate measles infections. Thiols and vitamins such as vitamin D are essential to cellular function and show promise in attenuating the hyperactive immune response causing post-viral complications such as multiple sclerosis [48].

Zinc is another essential element for a normal immune function. It contributes to the maturation and activity of immune cells, as well as to cytokine regulation, and has anti-inflammatory mechanisms. Given these properties, zinc has been used to support the treatment of a variety of acute and chronic diseases. It has been hypothesized that in measles patients who are zinc-deficient, zinc supplementation may reduce morbidity associated with measles [49]. In a study that reviewed the effectiveness of zinc supplementation on measles, 85 children with measles were observed. However, the results did not show a statistically significant difference in mortality between the children who received zinc and a placebo, nor was there a difference observed in the time to fever resolution. While zinc in theory may offer support, there is insufficient evidence to confirm or refute a benefit, and we thus suggest future studies to confirm if zinc can serve as an adjunct therapy for measles [50].

#### 4.6.3. Treatments

There is currently no FDA-approved antiviral therapy for measles. Management is primarily supportive, with adjunctive therapies used in select cases and complications of measles. Supportive care remains the foundation of measles management. This includes hydration, antipyretics, nutritional support, and treatment of secondary bacterial infections [51]. These have been shown to mitigate the disease severity, address complications, and support immune recovery. Although current treatments are supportive, many studies have continued to search for a potential antiviral therapy.

Ribavirin, a nucleoside analog, has demonstrated broad-spectrum activity against several RNA viruses. One experimental study evaluated ribavirin conjugated to gold nanoparticles (AuNPs) for its potential to improve antiviral effects. Vero cells, cells derived from a monkey’s kidney, and MTT assays were used to check the cell viability and toxicity. The study showed that ribavirin-AuNP complexes were not toxic, and ribavirin with AuNPs reduced measles by 78%, with the strongest antiviral effect occurring when added after infection. However, this remains a laboratory-based study and has yet to be proven clinically effective [52,53].

Another study evaluated the effect of ribavirin on the hospital stay of measles patients. The results showed that the stay was shorter in the ribavirin group (4 days) compared to the non-ribavirin group (6 days). However, the difference was not statistically significant, and ribavirin was also co-administered with other drugs and supplements, making it hard to isolate its effectivity [52].

Ribavirin has also been considered a possible antiviral therapy for severe complications such as SSPE. Ribavirin inhibits measles virus replication in neuronal cell models relevant to SSPE and is considered an emerging therapy. Although nucleoside analogs and antivirals are being explored and showing promise, the current evidence is limited, has small study sizes, and is not the clinical standard [53].

To treat severe complications such as encephalitis, high doses of corticosteroids, intravenous immunoglobulin, or plasmapheresis are sometimes also combined with antiviral treatment or vitamin A supplementation. Measles cause severe neurological complications beyond common respiratory and rash symptoms, so the management shifts from supportive to interventions such as high-dose glucocorticoids despite limited evidence for standardized therapy in this setting [54].

SSPE has multiple therapeutic approaches, but none are curative; instead, they may delay progression or provide symptomatic benefits. Immunomodulatory agents such as inosine dimepranol acedoben (also known as isoprinosine) are commonly used with or without ribavirin. In some cases, additional therapies, including intravenous immunoglobulin, intrathecal interferon alfa, and amantadine, are also administered [55,56].

A case report described the effects of remdesivir, a nucleoside analog, in SSPE, which caused a transient clinical improvement after the first two courses of remdesivir, while a third produced no further benefit. Although the patient eventually died, a post-mortem analysis revealed that no antiviral resistance mutations emerged during therapy. This led to a temporary rather than sustained improvement and is not established as an effective treatment, suggesting that further research is needed into more antiviral strategies for measles (Table 2) [57].

## 5. Preventative Measures

### 5.1. Prevention Rationale

Measles have been associated with preventable morbidity, mortality, and long-term immune dysfunctions, but as they have no targeted antiviral therapy, the emphasis remains on measures to prevent disease and transmission. Clinical management is largely supportive; therefore, prevention remains the most effective population-level control strategy. Due to their extreme transmissibility, measles can be seen in rapid resurgences when immunity gaps emerge, especially in countries with prior prevention and elimination. This leads to a failure in herd immunity and, as coverage dips, a disproportionate outbreak risk compared to other vaccine preventable diseases. Vaccination reduces transmission and indirectly protects vulnerable populations [58]. 

Measles prevention at the population level requires a community-wide immunity. Individual vaccination decisions directly affect vulnerable populations, and herd immunity is essential for measles control, as its prevention relies on achieving herd immunity though a high community vaccination coverage to protect those that cannot be vaccinated [59].

In a study analyzing the measles outbreak in the Kakumiro district, Uganda (February–May 2024), measles is shown in a global health context, highlighting the inadequate prevention measures that allow measles outbreaks to still occur where vaccine coverage is insufficient. Even with modern vaccines, measles outbreaks continue to occur when population immunity is insufficient, which emphasizes the ongoing importance of effective prevention strategies. These findings are generalizable to developed countries such as the U.S., showing that it is important even in developed countries to increase the routine coverage, catch-up campaigns, and targeted outreach in underserved areas [60].

### 5.2. Vaccination as Primary Prevention

Vaccination remains the primary source of prevention due to the virus’s extreme transmissibility and its capacity to induce prolonged immune dysfunction. The live-attenuated vaccine is administered as part of the MMR vaccine, offering primary prevention by inducing humoral and cellular immune responses [61]. Following vaccination, neutralizing antibodies directed against measles hemagglutinin protein prevent viral entry into host cells, while memory B- and T-cell responses confer long-term protection against subsequent exposures [62].

A two-dose vaccination strategy is required for effective population-level protection. As a single dose induces immunity in most recipients, some individuals fail to seroconvert due to factors such as maternal antibody interference or host immune variability [63]. As such, a second dose ensures seroconversion for primary non-responders and increased overall population immunity. A long-term immunogenicity study demonstrated that measles-neutralizing antibodies remain high for at least ten years following the second (and third) dose of MMR vaccine, with most maintaining protective long-term immunity. Although neutralizing antibody levels decline gradually, the effectiveness of the two doses shows retained seroprotection against measles, with no need for frequent boosting [64].

By increasing individual immunity, the two-dose strategies raise population-level immunity above the herd immunity threshold. In order to prevent measles outbreaks, it becomes more crucial to maintain a complete two-dose schedule to preserve herd immunity. Measles vaccination also provides indirect protection through herd immunity. Infants younger than the recommended vaccination age, individuals with a contraindication to the MMR live vaccine, and immunocompromised patients rely on a high community vaccination coverage to limit viral circulation. Thus, measles vaccination serves not only as an individual health intervention but also as a collective protective measure [65]. In addition to preventing acute clinical illness, vaccination protects against measles-associated immune suppression, thereby preventing immune depletion and long-term susceptibility to secondary infections.

### 5.3. Post-Exposure Prophylaxis

In addition to routine vaccination, post-exposure prophylaxis (PEP) serves as a secondary prevention to measles, especially in outbreak settings. The timing of PEP is critical due to the relatively long incubation period of measles, with an estimated mean incubation period of 10 days before fever onset and 14 days before rash development. This extended incubation period provides a unique intervention window that is not present for many other viral infections [66].

For immunocompetent individuals 6 months of age and older who are exposed to measles, the MMR vaccine can be given within 72 h of exposure. This can prevent infection of these individuals and provide active immunization after exposure, serving as an effective PEP strategy in immunocompetent and vaccine-eligible individuals [41,67].

For individuals where the live vaccination is contraindicated or when the window for vaccine administration has passed, immune globulin may be administered intramuscularly or intravenously within 6 days according to the CDC recommendations. This provides passive immunity and serves to reduce the disease severity and associated complications. These special populations including infants, pregnant individuals, and immunocompromised patients who are unable to receive the MMR vaccine and must depend on PEP as a protective measure to reduce morbidity and mortality [41]. The effectiveness of PEP further highlights the importance of early case identification and contact tracing, as intervention must occur before systemic viral dissemination and immune depletion.

### 5.4. Infection Prevention and Control Measures

Measles transmission occurs primarily through aerosolized particles and respiratory droplets. The virus can be inactivated by WHO-recommended alcohol-based hand rub formulations, oral rinses, and surface disinfectants. Consistent hygiene practices play an important role in limiting measles transmission in healthcare and community settings [68]. The measles virus can remain airborne for up to two hours, making negative-pressure rooms and adequate ventilation critical components of infection control. Proper airflow management is essential in a healthcare environment. Administrative control measures including early identification of suspected cases, triage protocols, and screening of visitors and healthcare personnel complement vaccination efforts by preventing infected individuals from exposing others [15].

### 5.5. Challenges to Effective Prevention

In the healthcare setting, many challenges arise that hinder the effective prevention of measles due to its high transmissibility and the increased exposure risk in emergency departments. Delayed care and a non-specific presentation can often result in missed measles diagnoses, further complicating the timely implementation of control measures in clinical settings. Patients may also have unclear immunization histories and lack systematic vaccination records, leading to an increased susceptibility and risk of nosocomial transmission. Failure to consistently use appropriate protective equipment and implement control measures further fuels outbreaks [69].

In addition to healthcare challenges, vaccination schedules can affect prevention efforts. Although most children who receive early vaccination seroconvert after the first dose, children vaccinated before 8.5 months of age exhibit faster antibody decay and loss of their protective neutralizing antibody levels within 6 years [70].

Despite substantial declines in measles incidence following vaccination programs, outbreaks continue to occur due to multiple factors, such as vaccine hesitancy, inadequate immunization coverage, and imported cases. One study focused on policies surrounding measles in developed countries such as the U.S. and Japan, including vaccine schedules, immunity assessments, and verification practices. Despite progress towards measles elimination, achieving a high vaccination coverage and vaccine hesitancy remain critical challenges. Continued measles resurgences and outbreaks emphasize the need for ongoing surveillance and a sustained coverage of measles. Additionally, variation in immunity assessment methods impacts the surveillance accuracy and control, suggesting the need to strengthen international collaboration and standardize assessment protocols to support policy optimization and an effective public health strategy [71].

To restore measles vaccination coverage, countries may implement supplementary immunization activities (SIAs), including mass vaccination campaigns and targeted catch-up efforts. These strategies have been shown to reduce an outbreak’s size and transmission by closing immunity gaps among individuals who have missed routine immunization schedules [72]. SIAs not only play a critical role in reaching under-immunized populations but also represent a high return on investment due to their cost-effectiveness. Further studies have shown that using multiple tactics to educate the public, such as providing incentives, utilizing technology and text messaging, and reducing vaccination prices, has led to improvements in the measles vaccination coverage and timeliness [73]. Overall, these findings underscore the importance of restoring the measles vaccination coverage, and they highlight the multicomponent strategies that countries can implement to prevent future outbreaks.

## 6. Conclusions

The resurgence of measles in the U.S. and globally represents a major public health challenge that extends beyond a simple viral infection. While traditionally viewed as a childhood illness, MeV is a sophisticated pathogen capable of evading the human immune response through the depletion of immunological memory. Complications such as these significantly increase long-term morbidity and mortality, making the virus more dangerous than some acute symptoms may suggest. While nutritional interventions like vitamin A supplementation and antioxidants show promise in reducing the severity, the lack of a targeted antiviral therapy necessitates a focus on prevention. Vaccination is the only definitive defense; the two-dose MMR vaccine is highly effective, yet even minor dips in coverage below the 95% herd immunity threshold led to exponential increases in outbreaks. Additionally, the recent spike in cases in 2024 and 2025 indicates that technical vaccine efficacy is insufficient if public mistrust persists.

Future research should prioritize the development of rapid-response antivirals and improved genetic screening for vaccine non-responders. Ultimately, eliminating measles requires a dual approach: advancing the molecular understanding of host–virus interactions and addressing the drivers of vaccine hesitancy to restore robust herd immunity.

## Figures and Tables

**Table 1:** Breakdown of immune system components that MeV affects as well as whether they are part of the innate or adaptive immune system and a summary of their primary role.

Innate or Adaptive	Cell Type	Primary Role
Innate	Dendritic cells	Detect foreign invaders and further transmit the infection to mononuclear cells such as thymocytes, B cells, and hematopoietic stem cells, which are responsible for dissemination to non-lymphoid organs.
Innate	RLRs	Sense viral infections and initiate transcription of IFN, leading to several downstream pathways that mediate inflammation.
Adaptive	Memory lymphocytes	Provide immunity to previously exposed pathogens to mount a stronger immune response after the next exposure.
Adaptive	MAIT cells	Subset of T-cells that are responsible for cytokine signaling, migration, and proliferative expansion. Make up 10% of blood T cells and 45% of liver T cells.

**Table 2:** Summary of supportive, adjunctive, and investigational supplementation and treatment strategies evaluated for measles and its complications, illustrating the limited evidence base beyond supportive care and vitamin A.

Intervention	Indication	Proposed Mechanism	Number of Studies	Number of Participants	Key Findings
Vitamin A	Acute measles (children < 5 years)	Restores retinoid homeostasis; stabilizes epithelial and immune function; mitigates inflammation	Numerous	Large, population-based	Reduces measles-related morbidity and mortality; benefit extends beyond correction of deficiency
Glutathione/Thiol	Acute measles; SSPE	Reduces mitochondrial reactive oxygen species; counteracts virus-induced oxidative stress	1 study	30	Oxidative stress markers altered in measles and SSPE; proposed as adjunctive therapy
Vitamin D	Acute measles	Modulates immune response; reduces oxidative stress	1 study	5681	Inverse association between vitamin D levels and measles antibody titers; therapeutic role remains theoretical
Zinc	Acute measles (children)	Supports innate and adaptive immune function	1 trial	85 children	No statistical significance in mortality or fever resolution; insufficient evidence for routine use
Supportive	All measles cases	Symptom control; prevention of complications	Widespread	Not applicable	Foundation of measles management; mitigates severity and supports recovery
Ribavirin (in vitro)	Experimental measles treatment	Inhibition of viral RNA replication	1 study	Cell culture (Vero cells)	Ribavirin–AuNP complexes reduced measles replication by ~78%; non-toxic in vitro
Ribavirin (clinical)	Hospitalized measles cases	Antiviral activity	1 study	100	Shorter hospital stay (4 vs. 6 days), not statistically significant; confounded by co-administration
Corticosteroid/IVIG/plasmapheresis	Measles encephalitis	Immunomodulation	1 study	1	Used for severe neurologic complications; no standardized regimen
Isoprinosine (inosine dimepranol acedoben)	SSPE	Immunomodulatory and antiviral effects	Multiple	Small cohorts	May delay disease progression or provide symptomatic benefit; not curative
Remdesivir	SSPE	Nucleoside analog inhibiting measles virus RNA-dependent RNA polymerase	1 report	1	Transient clinical improvement after initial courses; no sustained benefit

## Data Availability

No new data were created or analyzed in this study.

## Abbreviations

The following abbreviations are used in this manuscript:

MeV	Measles virus
SSPE	Subacute sclerosing panencephalitis
CDC	Center for Disease Control
WHO	World Health Organization
